# Circulating microRNAs as diagnostic biomarkers for melanoma: a systematic review and meta-analysis

**DOI:** 10.1186/s12885-023-10891-6

**Published:** 2023-05-08

**Authors:** Nan Wu, Hongyan Sun, Qian Sun, Fangqing Zhang, Lingli Ma, Yue Hu, Xianling Cong

**Affiliations:** 1 Department of Dermatology, China-Japan Union Hospital of Jilin University, Xiantai Road 126, Changchun, Jilin 130033 PR China; 2 Department of Biobank, China-Japan Union Hospital of Jilin University, Changchun, Jilin PR China; 3 Department of Endocrinology and Metabolism, China-Japan Union Hospital of Jilin University, Changchun, Jilin PR China

**Keywords:** Melanoma, Circulating microRNA, Diagnostic, Biomarkers, Meta-analysis

## Abstract

**Background:**

Recent studies have shown that circulating microRNAs (miRNAs) can be used as diagnostic biomarkers for melanoma. This study aimed to evaluate the diagnostic value of circulating miRNAs for melanoma.

**Methods:**

A comprehensive literature search was conducted and the quality of the included literature was evaluated using QUADAS-2 (Quality Assessment for diagnostic accuracy studies), and the diagnostic accuracy was assessed by pooled sensitivity, specificity, positive likelihood ratio (PLR), negative likelihood ratio (NLR), diagnostic odds ratio (DOR), and area under the curve (AUC). We used Deeks’ funnel plot to evaluate publication bias.

**Results:**

The meta-analysis included 10 articles covering 16 studies, and the results showed that circulating miRNAs provide high diagnostic accuracy for melanoma. The overall pooled sensitivity was 0.87 (95% CI: 0.82–0.91), specificity was 0.81 (95% CI: 0.77–0.85), PLR was 4.6 (95% CI: 3.7–5.8), NLR was 0.16 (95% CI: 0.11–0.23), DOR was 29 (95% CI: 18–49), and AUC was 0.90 (95% CI: 0.87–0.92), respectively. Subgroup analysis showed better diagnostic value in miRNA clusters, European population, plasma miRNAs, and upregulated miRNAs compared to other subgroups.

**Conclusions:**

The results indicated that circulating microRNAs can be used as a non-invasive biomarker for the diagnosis of melanoma.

**Supplementary Information:**

The online version contains supplementary material available at 10.1186/s12885-023-10891-6.

## Introduction

Melanoma is a fatal and aggressive tumor, as it is associated with poor prognosis in patients with advanced or metastatic disease [1–4]. Over the past decade, the incidence of occurrence melanoma has increased every year [5–8]. A worldwide total of 325 000 new melanoma cases and 57 000 deaths was estimated for 2020, if 2020 rates continue, the burden from melanoma is estimated to increase to 510 000 new cases (a roughly 50% increase) and to 96 000 deaths (a 68% increase) by 2040 [9]. Early-stage melanoma can be effectively controlled by surgical resection. However, melanoma is highly susceptible to metastasis, and there is no effective treatment for patients with metastasis or diffusion in the late stages of the disease, for patients with III/IV melanoma the overall 5-year survival may be less than 10% [10, 11]. Although early diagnosis is very important for treatment, pathological diagnosis is currently the only diagnostic tool. Hence, patients with melanoma are prone to missing the opportunity for early diagnosis and treatment [12, 13]. This necessitates investigating simple, less invasive, and reliable biomarkers that improve the efficiency of early diagnosis and clinical prognosis of melanoma patients.

MicroRNAs (miRNAs) are a class of small, short-stranded non-coding RNAs, which are approximately 19–24 nucleotides in length and are found in eukaryotes [14, 15]. miRNAs are often dysregulated in human tumors and contribute to the development and progression of cancer [16]. Circulating microRNAs consist of a variety of microRNAs that are present in various extracellular fluids (e.g., plasma, serum, and whole blood) and which may be released passively during apoptosis or lysis or actively by the surviving tissue cells [17]. Circulating microRNAs exist stably in the circulatory system bound to proteins to avoid catabolism, and can be used as biomarkers for early diagnosis, therapeutic response, and prognosis of various human diseases [18–20]. In recent years, several studies have demonstrated the potential of circulating microRNAs as biomarkers in the early diagnosis of melanoma, but evidence-based data is still lacking [21–24]. In 2010, Leidinger et al. showed that 16 significantly downregulated miRNAs in blood cells can be used as biomarkers of melanoma [25]. Subsequently, multiple miRNAs in plasma or serum were successively identified as having diagnostic value for melanoma, including miR-320a-5p, miR-21-5p, miR-210-3p, miR-185-5p, miR-221-3p, miR-1246d [26–33]. In addition, several studies have shown that circulating miRNAs panels have better diagnostic efficacy for melanoma, such as the combination of miR-149-3p, miR-150-5p and miR-193a-3p [34, 35]. However, studies on the diagnostic efficacy of these circulating miRNAs for melanoma have been inconclusive. Therefore, we conducted a meta-analysis aiming to explore the diagnostic value of circulating miRNAs as a non-invasive biomarker for the detection of melanoma.

## Materials and methods

This meta-analysis followed the Preferred Reporting Items for Systematic Reviews and Meta-analyses of Diagnostic Test Accuracy (PRISMA-DTA) and the PRISMA 2020 declaration [36–38].

**Fig. 1:**
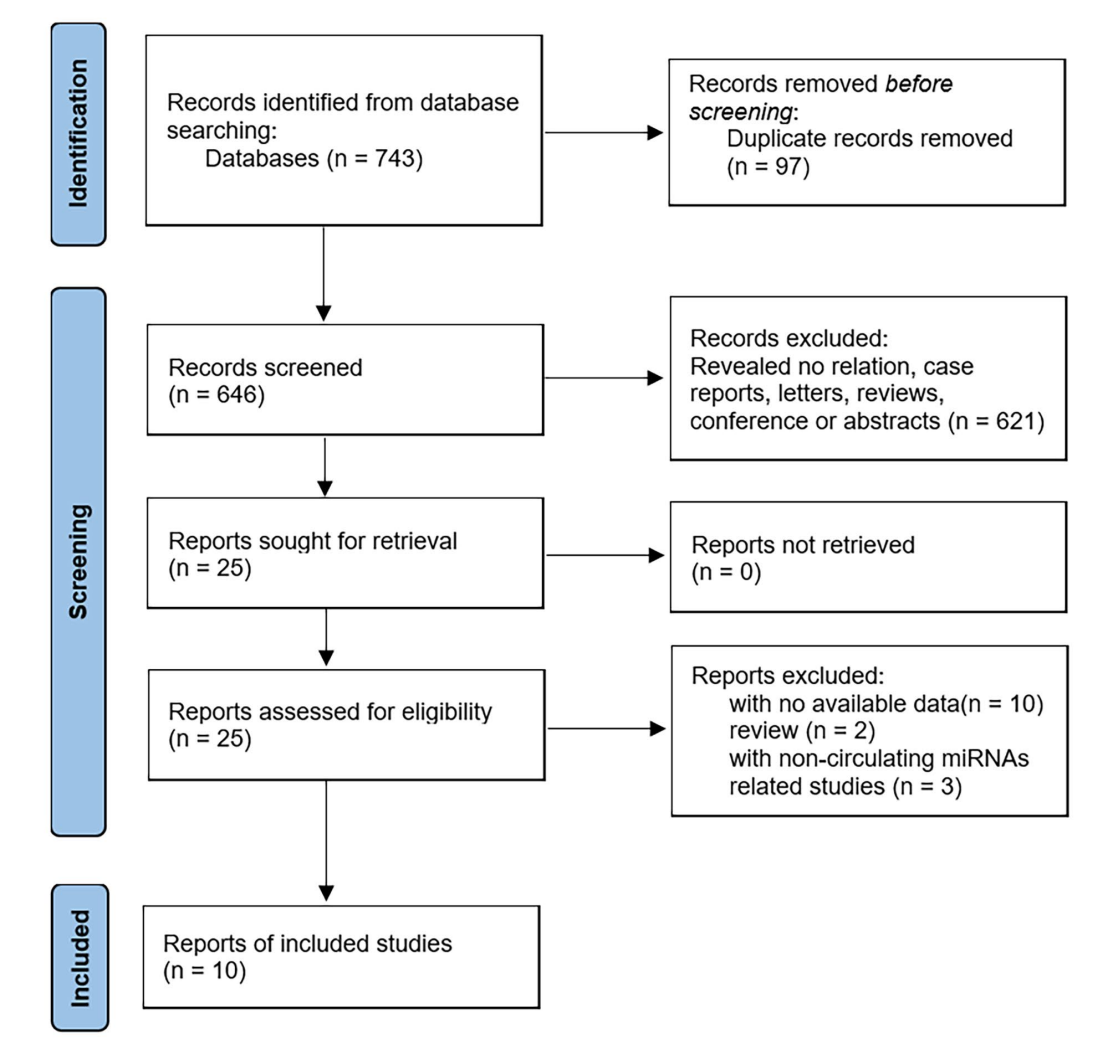
flow diagram for the study selection process

### Search strategy and data sources

We searched for articles in four electronic databases including Web of Science, PubMed, China National Knowledge Infrastructure, and Wanfang. All the English and Chinese publications until December 25, 2022 were searched without any restriction to countries or article type. A reference list of all selected articles was independently screened to identify additional studies left out in the initial search. The search terms used were “melanoma” and (“microRNA” or “miRNA” or “miR”) and (“circulating” or “circulation” or “serum” or “plasma”) and (“sensitivity” or “specificity” or “predictive” or “accuracy”). Two independent reviewers used the above search criteria and pre-defined inclusion and exclusion criteria to evaluate available literature. In addition, a manual search of relevant articles and references cited in these articles was conducted to identify all available studies. PROSPERO Registration Number is CRD42022320573.

**Table 1:** Characteristics of the included studies

Study	Country	miRNA	Mode	Sample size			Type of sample	Time	Method	Reference	Cut-off value	Sen	Spe
				**Melanoma** (M/F)	**Control** (M/F)	**Metastatic melanoma**							
Leidinger, [25]	Germany	miRNA clusters (16)	up	35 (20/15)	20 (7/13)	4(11.4%)	Blood	NA	qRT-PCR	RNU48	NA	98.9%	95.0%
Stark, [51]	Australia, Germany	miRNA clusters (7)	NA	119 (76/43)	130 (59/71)	119(100%)	Serum	Other	qRT-PCR	RNU6	NA	93.0%	82.0%
Guo, [21]	China	miR-16	down	120 (53/67)	120 (65/55)	60(50%)	Serum	NA	qRT-PCR	Cel-miR-39	0.585	80.0%	71.7%
Yao, [52]	China	miRNA clusters (4)	up	12 (5/7)	20 (9/11)	7(58.3%)	Serum	Before	qRT-PCR	NA	NA	83.3%	85.0%
Armand-Labit, [29]	France	miRNA clusters (2)	up	31 (17/14)	43 (24/19)	31(100%)	Plasma	Before	qRT-PCR	NA	NA	90.5%	89.1%
Bai, [49]	China	miR-10b	up	85 (45/40)	30 (16/14)	35(41.2%)	Serum	Before	qRT-PCR	U6 snRNA	NA	76.0%	88.0%
Fogli, [34]	Italy	miRNA clusters (3)	up/down	30 (17/13)	32 (18/14)	16(53.3%)	Plasma	Other	qRT-PCR	U6 snRNA	NA	94.8%	83.9%
		miR-149-3p	up									93.3%	87.5%
		miR-150-5p	up									96.7%	70.0%
		miR-193a-3p	down									76.7%	62.5%
		miR-15b-5p	up									90.0%	77.4%
		miR-524-5p	down									90.0%	68.8%
Van laar, [35]	Australia	miRNA clusters (28)	NA	35(NA)	22(NA)	NA	Blood	Other	microarray	NA	NA	86.0%	NA
Mo, [50]	China	miR-21	up	60 (30/30)	40 (20/20)	45(75.0%)	Serum	NA	qRT-PCR	U6	4.841 0.451	77.8%	82.2%
		miR-489	down									75.6%	80.0%
Li, [53]	China	miR-21	up	121 (78/43)	121 (76/45)	34(28.1%)	Plasma	Before	qRT-PCR	U2 snRNA	NA	77.6%	87.0%

**Abbreviations**: M: male; F: female; Sen: sensitivity; Spe: specificity; Time: samples collection time; Before: before surgery and treatment; NA: not available

**Fig. 2:**
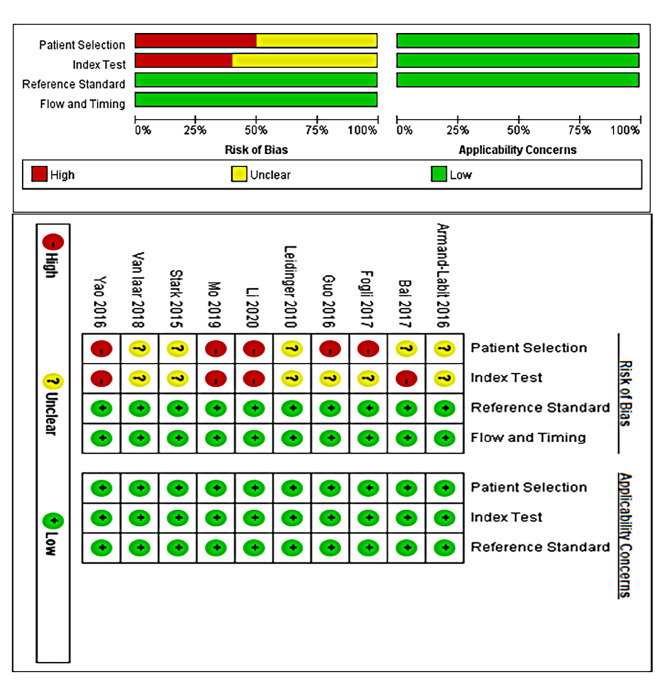
Risk of bias analyses

### Inclusion and exclusion criteria

The inclusion criteria encompassed the following criteria: (1) Human studies investigating circulating miRNAs as a method for diagnosis of melanoma patients in comparison with healthy individuals or cancer-free patients and (2) Sensitivity, specificity, and sample size data were used for calculating the value of true positives (TP), false positives (FP), false negatives (FN) and true negatives (TN). The exclusion criteria were as follows: (1) Duplicates; (2) Reviews; (3) Letters; (4) Conference abstracts; and (5) Studies that were irrelevant to the diagnosis of melanoma.

**Fig. 3:**
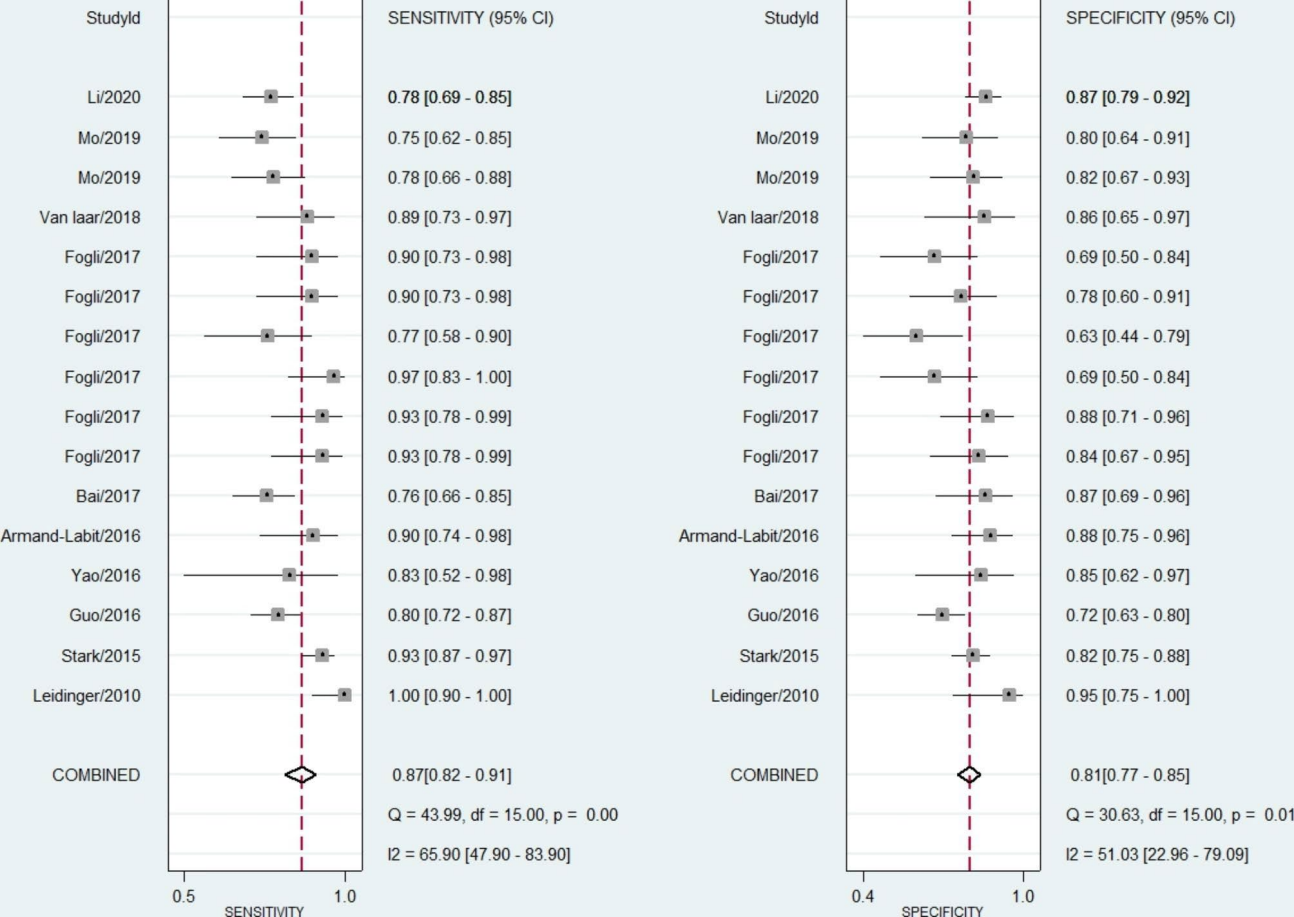
Forest plot pool sensitivity and specificity of circulating miRNAs in diagnosis of melanoma

### Study sections and data extraction

Two researchers separately reviewed all the eligible studies and extracted the following information: first author name, publication year, country, miRNA profile, regulation mode(up/down), sample size (number of melanoma patients, controls and metastatic melanoma patients), sample type, sample collection time, method, sensitivity, specificity, TP, FP, FN and TN. All disagreements were resolved by consulting a third author to reach a consensus while avoiding any bias.

**Fig. 4:**
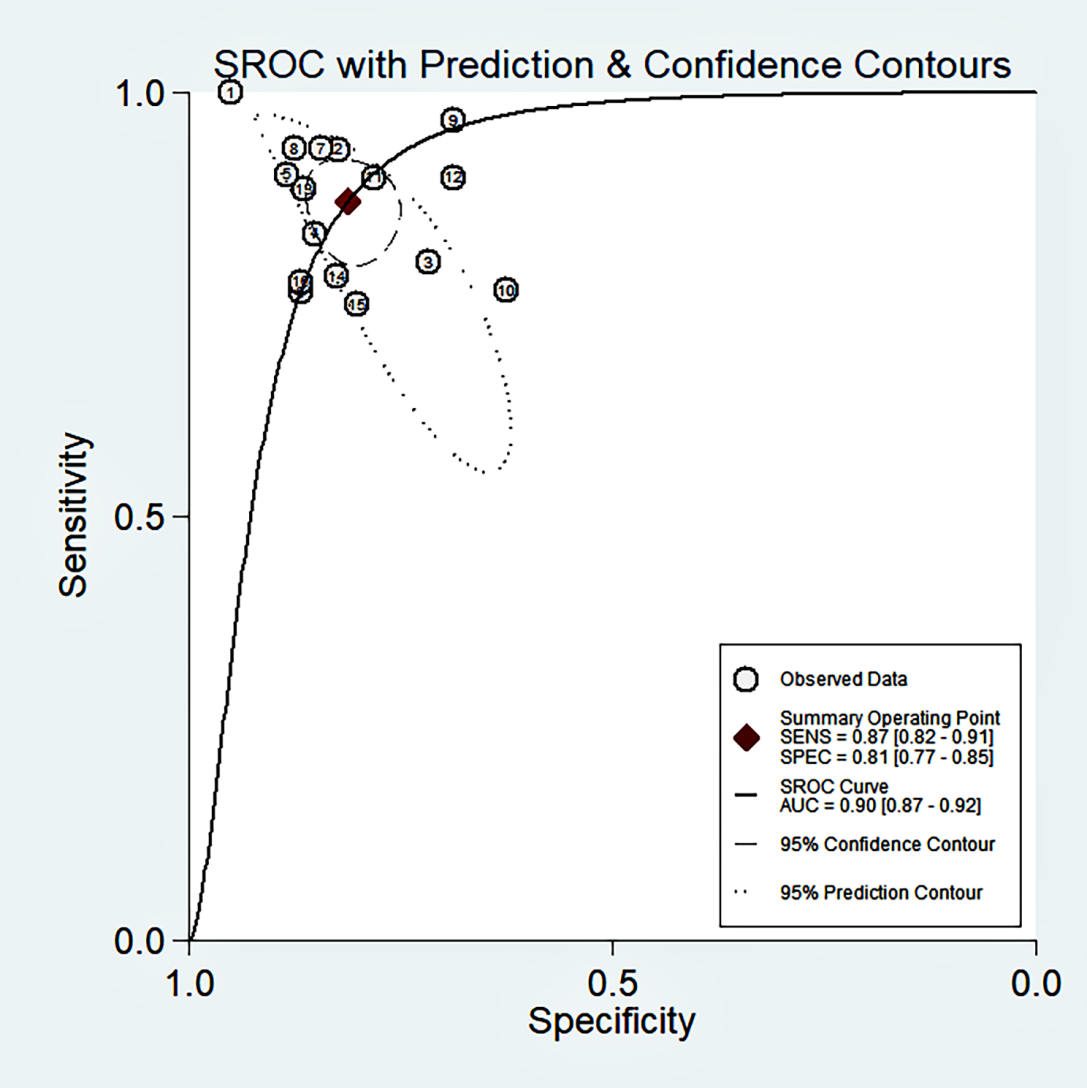
SROC curve of diagnostic power of circulating miRNAs for melanoma

### Quality assessment

Two independent researchers evaluated the included studies according to the Quality Assessment of Diagnostic Accuracy 2 (QUADAS-2) checklist [39]. This checklist composed of four domains i.e., Patient Selection, Index Test, Reference Standard, and Flow and Timing. The clinical applicability of selected patients, index testing, and standard of reference were evaluated respectively. Significant issues included “yes,” “no,” and “uncertainty.” Risk level of bias were divided into “high”, “low”, or “uncertainty”. Any disagreement was discussed and then resolved by consensus.

**Table 2:** Summary estimates of subgroup analysis for diagnostic test accuracy

Subgroups	Number of studies	Sen (95%CI)	Spe (95%CI)	PLR (95%CI)	NLR (95%CI)	DOR (95%CI)	AUC (95%CI)
Overall	16	0.87(0.82, 0.91)	0.81(0.77, 0.85)	4.6(3.7, 5.8)	0.16(0.11, 0.23)	29(18, 49)	0.90(0.87, 0.92)
**Area**							
Europe	10	0.92(0.88, 0.95)	0.80(0.73, 0.86)	4.7(3.3, 6.8)	0.09(0.05, 0.16)	51(22, 117)	0.94(0.91, 0.95)
Asia	6	0.78(0.73, 0.82)	0.82(0.75, 0.87)	4.2(3.1, 5.8)	0.27(0.23, 0.32)	16(10, 24)	0.81(0.77, 0.84)
**Mode**							
up	9	0.89(0.80, 0.94)	0.84(0.80, 0.88)	5.6(4.4, 7.2)	0.14(0.08, 0.24)	41(21, 78)	0.88(0.85, 0.91)
down	4	0.80(0.74, 0.84)	0.71(0.65, 0.77)	2.8(2.2, 3.5)	0.29(0.22, 0.37)	10(6, 15)	0.82(0.79, 0.86)
**Methods**							
qRT-PCR	15	0.87(0.82, 0.91)	0.81(0.76, 0.85)	4.6(3.6, 5.8)	0.16(0.11, 0.23)	29(17, 49)	0.90(0.87, 0.92)
microarray	1	0.89	0.86	/	/	/	/
**Reference**							
U6	10	0.87(0.81, 0.92)	0.79(0.74, 0.83)	4.1(3.3, 5.2)	0.16(0.11, 0.25)	25(14, 44)	0.86(0.82, 0.88)
others	6	0.88(0.78, 0.94)	0.86(0.77, 0.92)	6.3(3.6, 11.2)	0.14(0.07, 0.28)	44(14, 136)	0.93(0.91, 0.95)
**Collection time**	13						
before	4	0.79(0.74, 0.84)	0.87(0.82, 0.91)	6.0(4.3, 8.6)	0.24(0.19, 0.31)	25(15, 42)	0.90(0.87, 0.93)
other	9	0.91(0.87, 0.94)	0.79(0.73, 0.84)	4.4(3.3, 5.8)	0.12(0.08, 0.17)	38(20, 71)	0.93(0.90, 0.95)
**Cut-off value**							
with cut-off value	3	0.78	0.78	/	/	/	/
without cut-off value	13	0.89(0.84, 0.93)	0.82(0.77, 0.86)	5.0(3.9, 6.5)	0.13(0.08, 0.20)	39(22, 69)	0.91(0.88, 0.93)
**miRNAs profile**							
Single	10	0.81(0.77, 0.85)	0.78(0.72, 0.83)	3.7(2.9, 4.7)	0.24(0.20, 0.29)	15(11, 21)	0.86(0.83, 0.89)
Cluster	6	0.93(0.89, 0.95)	0.85(0.80, 0.89)	6.2(4.6, 8.3)	0.09(0.06, 0.13)	73(41, 149)	0.95(0.93, 0.97)
**Type**							
serum	6	0.81(0.75, 0.86)	0.81(0.76, 0.86)	4.4(3.3, 5.8)	0.23(0.17, 0.32)	19(11, 31)	0.88(0.85, 0.90)
plasma	8	0.92(0.87, 0.95)	0.81(0.74, 0.86)	4.7(3.4, 6.5)	0.10(0.06, 0.17)	46(24, 86)	0.93(0.91, 0.95)
blood	2	0.93	0.90	/	/	/	/
**Sample size**							
≥ 100	6	0.81(0.75, 0.86)	0.81(0.76, 0.86)	4.4(3.3, 5.8)	0.23(0.17, 0.32)	19(11, 31)	0.88(0.85, 0.90)
<100	10	0.91(0.86, 0.95)	0.81(0.74, 0.87)	4.8(3.4, 6.9)	0.11(0.06, 0.18)	45(21, 99)	0.94(0.91, 0.95)

**Abbreviations**: Sen: sensitivity; Spe: specificity; PLR: positive likelihood ratios; NLR: negative likelihood ratios; DOR: diagnostic odds ratio; AUC: area under the curve; CI: confidence interval

**Fig. 5:**
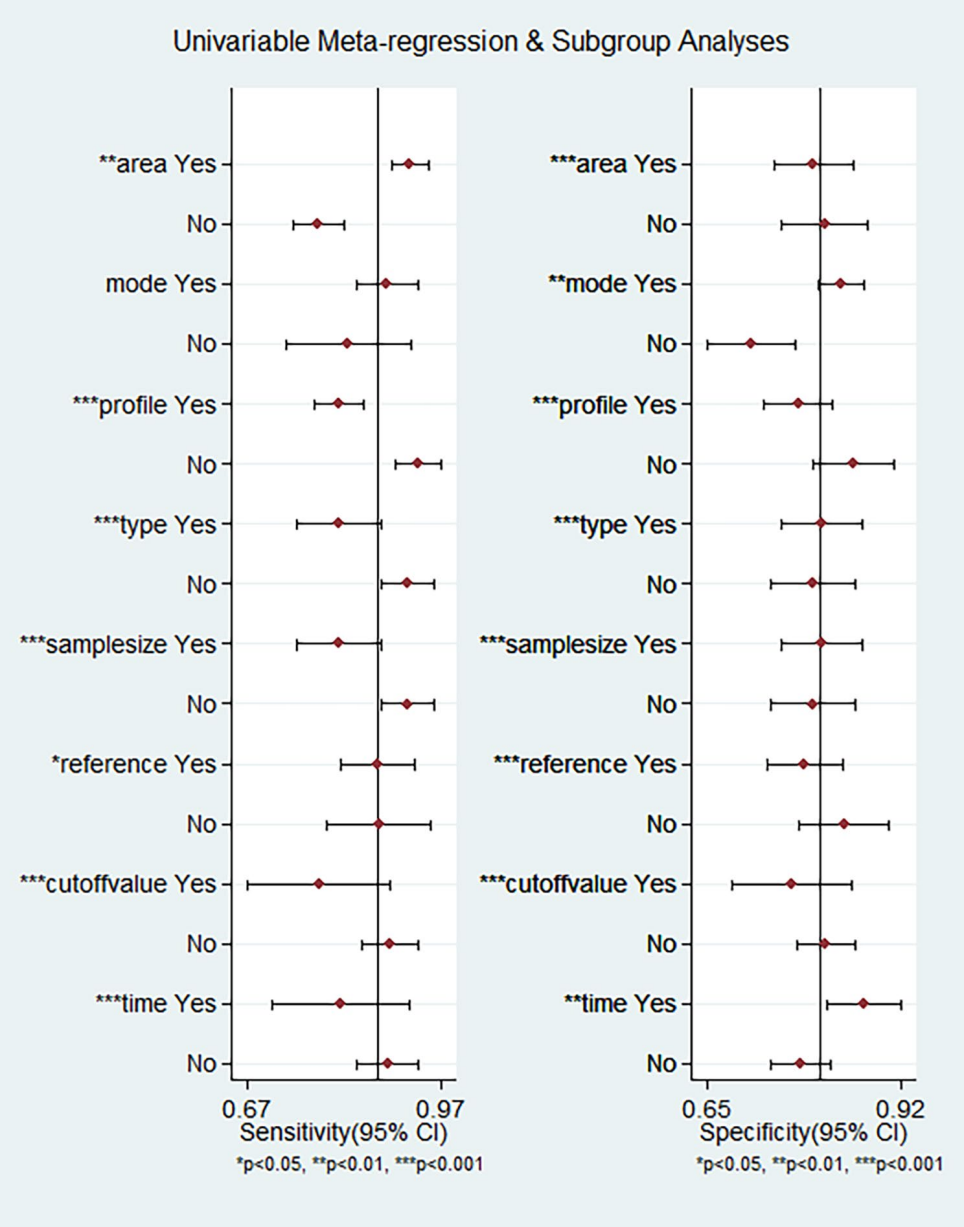
Univariable Meta-regression and Subgroup Analyses for diagnosis of miRNAs in melanoma

### Data analysis

We used STATA SE 14.0 (StataCorp, College Station, Texas, USA) and Revman 5.4 (Revman, the Cochrane Collaboration) to conduct the diagnostic meta-analysis. If the values of TP, FP, FN, and TN were not directly obtained, we calculated them based on the sample size of two groups, sensitivity, and specificity. Subsequently, the pooled sensitivity, specificity, positive likelihood ratio (PLR), negative likelihood ratio (NLR), diagnostic odds ratio (DOR), area under the curve (AUC), cut-off value, and 95% confidence intervals (95% CIs) were calculated. The AUC and DOR of the summary receiver characteristic curve (SROC) were used in assessing the overall performance of each diagnostic test. The Bi-variate mixed model was used to fitted for estimating the SROC curve. The heterogeneity was evaluated by using the index (I^²^). If the heterogeneity was determined to be significant (I^²^>50%), subgroup analyses and meta-regression analyses were conducted to investigate the main sources of heterogeneity, including area, sample size, sample type, miRNA profiling, methods, reference, cut-off value, regulation mode and stages. Additionally, the Fagan plot was employed to evaluate the clinical utility of miRNAs in distinguishing melanoma patients from controls.

**Fig. 6:**
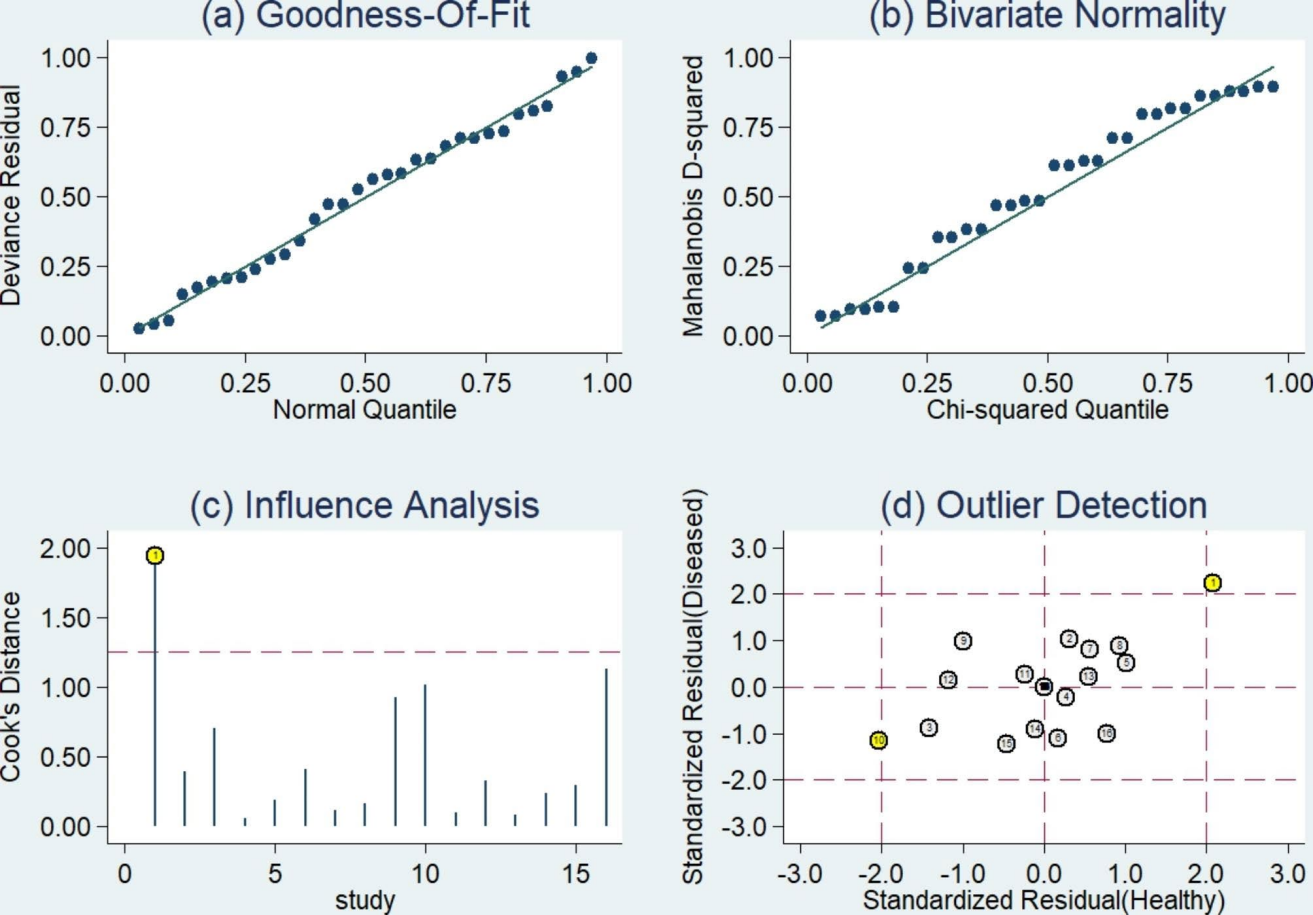
Sensitivity analysis (a) Goodness of fit; (b) Bivariate normality; (c) Influence analysis; (d) Outlier detection

**Fig. 7:**
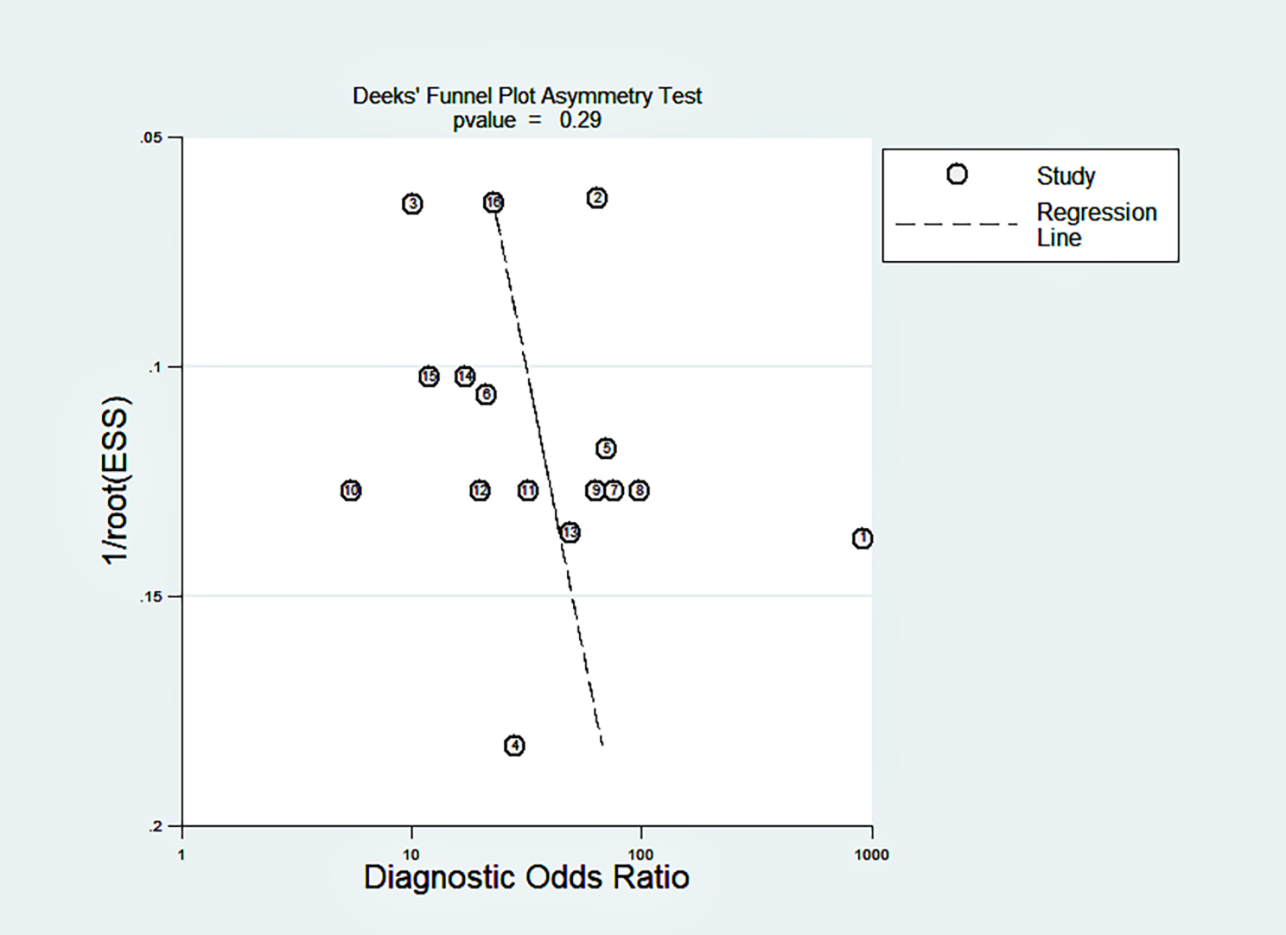
Deeks’ funnel plot for publication bias

**Fig. 8:**
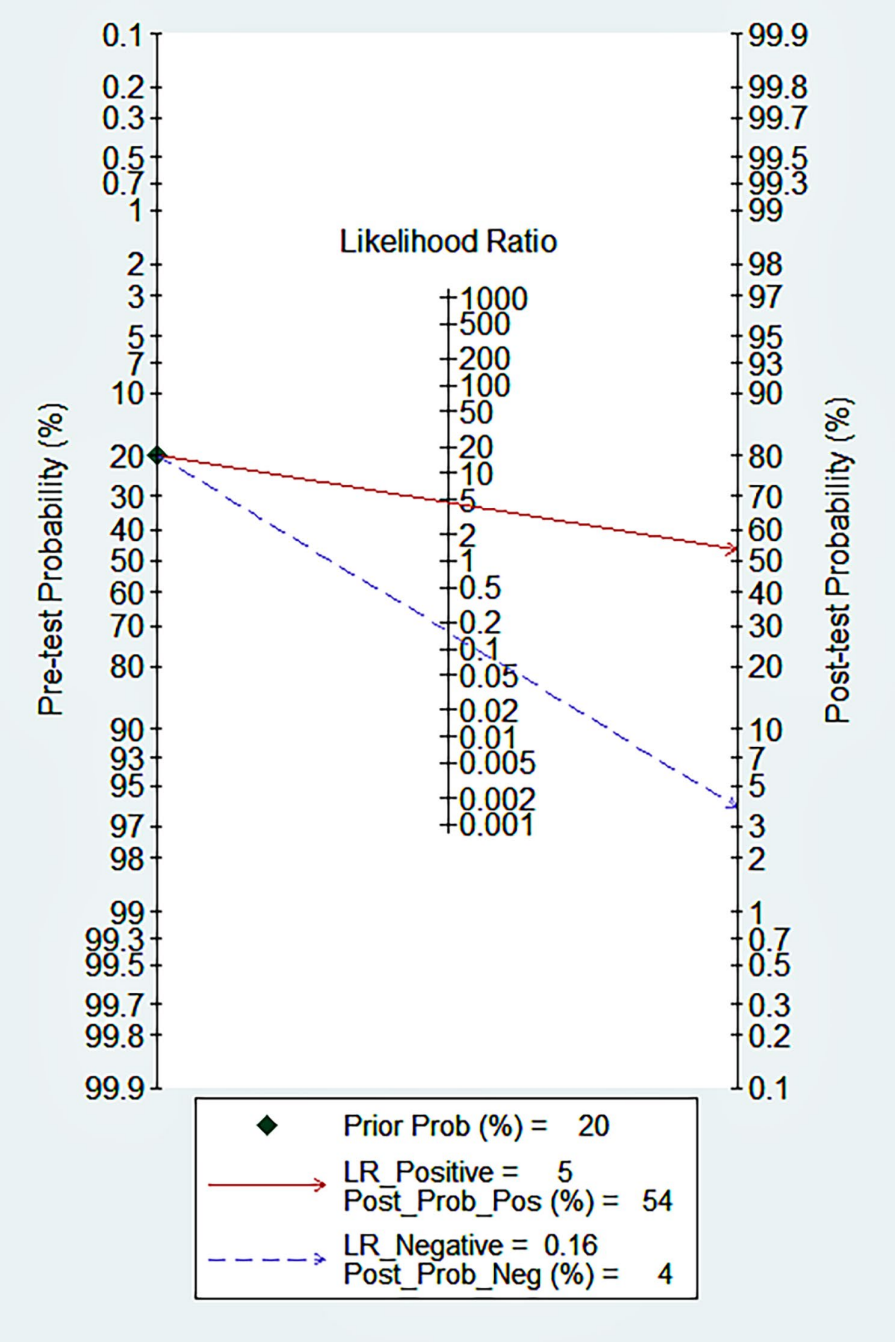
Fagan’s nomogram clinical applicability of circulating miRNAs in diagnosis of melanoma

## Results

### Study selection and characteristics

A literature search was conducted through the mentioned databases, and 743 articles were initially searched, of which 646 articles were identified after removing duplicates. Irrelevant articles, reviews, abstracts, and case reports were excluded by screening the titles and abstracts, and thereby excluding 621 articles. Further, 25 articles were fully read and evaluated, 10 of which were excluded because there did not contain enough available diagnostic data, [22, 26, 27, 30, 32, 40–44] another 2 were excluded because they were reviews, [45, 46] and 3 were excluded because they did not discuss circulating miRNAs [33, 47, 48]. Finally, a total of 16 studies from 10 publications met the inclusion criteria and were included in this meta-analysis (Fig. 1) [21, 25, 29, 34, 35, 49–53].

The basic characteristics of the included literature are shown in Table 1. Ten papers were published from 2010 to 2020. Among them, three used plasma samples, [29, 34, 35, 53] five used serum samples, [21, 49–52] and two used a blood cell sample, [25, 35] containing a total of 648 melanoma samples and 578 control samples. There were 351 metastatic melanoma patients, except for one study that did not report whether the patient with metastatic [35]. Seven studies have reported collection times for blood samples, including four were before surgery and treatment, [29, 49, 52, 53] and three were at the time of diagnosis and after surgery [34, 35, 51]. Five of these papers were from China, [21, 49, 50, 52, 53] two were from Germany, [25, 51] one was from Italy, [34] two were from Australia, [35, 51] and one was from France [29]. Six studies analyzed miRNAs clusters, [25, 29, 34, 35, 51, 52] and the other 10 studies assessed single miRNAs, [21, 34, 49, 50, 53] miRNAs expression was upregulated in nine studies, [25, 29, 34, 49, 50, 52, 53] downregulated in four studies, [21, 34, 50] both upregulated and downregulated in one study, [34] and not specified in two study [35, 51]. One study detected the expression level of miRNA by microarray method, [35] while other studies detected the expression level of miRNA by quantitative real-time chain reaction polymerase chain reaction (qRT-PCR), revealing the corresponding sensitivity and specificity. We then calculated TP, FP, FN, and TN in combination with the sample sizes.

### Quality assessment

Overall, the quality of the included literature was average, and most articles were case-control studies in which patients with confirmed melanoma were selected as subjects, and patients with suspected or unconfirmed diagnosis were excluded. This may have resulted in an overestimation of the diagnostic accuracy. Most studies interpreted the index test results with knowledge of the results of the reference standard, which may have affected the interpretation of the results and caused potential bias. The quality of the included literature is shown in Fig. 2.

### Analysis of circulating miRNAs diagnostic accuracy in melanoma

The forest plot of the pooled sensitivity and specificity of circulating miRNAs in the diagnosis of melanoma is shown in Fig. 3. By pooling the forest plots, we found significant heterogeneity (I^2^ > 50%, p ≤ 0.01) in the analysis results. The combined estimation of the diagnostic accuracy of circulating miRNAs in melanoma was as follows (Table 2): sensitivity: 0.87 (95% CI: 0.82, 0.91); specificity, 0.81 (95% CI: 0.77, 0.85); PLR, 4.6 (95% CI: 3.7, 5.8); NLR, 0.16 (95% CI: 0.11, 0.23); DOR, 29 (95% CI: 18, 49). The SROC curve did not show a typical “shoulder-arm-like” distribution (Fig. 4), suggesting that there is no threshold effect in this study. The AUC was 0.90 (95% CI: 0.87, 0.92), which suggests that the overall miRNAs have outstanding diagnostic accuracy.

### Subgroup analysis and meta-regression

To find sources of heterogeneity among studies, we performed subgroup analysis and meta-regression analysis based on the study area, detection method, reference, cut-off value, miRNA regulation mode, miRNA profiling, sample type, and size (Table 2; Fig. 5). In regression analysis, we found that specificity was influenced by the area, miRNA regulation mode, miRNA profiling, sample type, and size. The sensitivity was affected only by area, mode, miRNA profiling, sample type, reference, and size. In subgroup analysis, we found that the studies performed on a European population showed better diagnostic accuracy than those conducted on an Asian population (sensitivity 0.92 vs. 0.78, specificity 0.80 vs. 0.82, PLR 4.7 vs. 4.2, NLR 0.09 vs. 0.27, DOR 51 vs. 16, and AUC 0.94 vs. 0.81). Compared with single miRNA, the diagnostic accuracy of miRNA clusters was shown to have a higher overall diagnostic accuracy (sensitivity 0.81 vs. 0.93, specificity 0.78 vs. 0.85, PLR 3.7 vs. 6.2, NLR 0.24 vs. 0.09, DOR 15 vs. 73, and AUC 0.86 vs. 0.95). In addition, the studies that indicated upregulation of miRNAs demonstrated a higher diagnostic accuracy than those that reported downregulation of miRNAs (sensitivity 0.89 vs. 0.80, specificity 0.84 vs. 0.71, PLR 5.6 vs. 2.8, NLR 0.14 vs. 0.29, DOR 41 vs. 10, and AUC 0.88 vs. 0.82). Further, miRNAs in the plasma samples showed a higher diagnostic accuracy than those in the serum samples (sensitivity 0.92 vs. 0.81, specificity 0.81 vs. 0.81, PLR 4.7 vs. 4.4, NLR 0.10 vs. 0.23, DOR 46 vs. 19, and AUC 0.93 vs. 0.88). Additionally, there are significant difference in diagnostic accuracy between the subgroups with sample sizes (> 80 vs. <80: sensitivity 0.81 vs. 0.91, specificity 0.81 vs. 0.81, PLR 4.4 vs. 4.8, NLR 0.23 vs. 0.11, DOR 19 vs. 45, and AUC 0.88 vs. 0.94) and no significant difference in the internal reference (U6 vs. others: sensitivity 0.87 vs. 0.88, specificity 0.79 vs. 0.86, PLR 4.1 vs. 6.3, NLR 0.16 vs. 0.14, DOR 25 vs. 44, and AUC 0.86 vs. 0.93). The diagnostic efficacy of collecting samples at other times may be better in the group than in the group before surgery and treatment (sensitivity 0.91 vs. 0.79, specificity 0.79 vs. 0.87, PLR 4.4 vs. 6.0, NLR 0.12 vs. 0.24, DOR 38 vs. 25, and AUC 0.93 vs. 0.90). However, the number of microarray, with cut-off value and blood sample studies subgroups is too small to be summarized and analyzed.

### Sensitivity analysis, publication bias test and clinical value

The sensitivity analysis is shown in Fig. 6. The goodness-of-fit and bivariate normal analysis showed that the bivariate mixed-effects model was robust for meta-analysis (Fig. 6a, b). Furthermore, outlier detection suggests that two studies [25, 34] may be the cause of heterogeneity (Fig. 6c, d). After excluding outliers, we found that there was no significant change in overall sensitivity (0.87 vs. 0.86), specificity (0.81 vs. 0.81), PLR (4.6 vs. 4.3), NLR (0.16 vs. 0.17), DOR (29 vs. 27), and AUC (0.9 vs. 0.89), indicating that the sensitivity of the included studies was low, and the results were more robust and credible. A Deeks’ funnel plot was drawn to assess publication bias (Fig. 7), and we found that t = 1.11 and p = 0.29, indicating that no significant publication bias existed in the included studies. The Fagan diagram (Fig. 8) for evaluation indicated that the post-test probability was 54%, showing that the probability of melanoma patients diagnosed with melanoma increased from 20 to 54% after using circulating microRNA. The NLR was 0.16 (95% CI 0.11–0.23) and the Fagan plot evaluation showed a post-test probability of 4%, indicating that the probability of melanoma-negative patients being diagnosed with melanoma after detecting a significant level of circulating microRNA in their measurements dropped from 50 to 4%.

## Discussion

In this meta-analysis, we systematically evaluated and analyzed 16 studies from 10 papers containing 648 melanoma and 578 control patients. The results showed that circulating miRNAs demonstrated high accuracy in the diagnosis of melanoma with sensitivities, specificities, and AUCs of 87%, 81%, and 0.90, respectively. In addition, we evaluated the clinical applicability of circulating miRNAs for detecting melanoma. The combined effect size results showed the PLR of circulating miRNAs in the diagnosis of melanoma was 4.6 (95% CI 3.7–5.8) and the pre-test probability was 20%. The DOR is another indicator used to judge the test performance. The larger the DOR value, the greater the diagnostic efficacy will be [54]. A DOR value greater than 1 indicates a better diagnostic test. The DOR was 29, indicating that circulating miRNAs can effectively distinguish malignant melanoma patients from control populations. The results demonstrate that the use of circulating microRNAs significantly improves the clinical diagnostic value of melanoma.

Subgroup analysis showed that the diagnostic efficacy of circulating miRNA in Europe (Sen: 92%, DOR: 51, AUC: 0.94) was significantly higher than that in Asia (Sen: 92%, DOR: 51, AUC: 0.94), which may be due to the epigenetic factors and genetic differences in the incidence of malignant melanoma in different races and regions [55]. The upregulated circulating miRNAs (Sen: 89%, Sen: 84%, DOR: 41, AUC: 0.88) in melanoma has better diagnostic efficacy than the downregulated miRNAs(Sen: 80%, Sen: 71%, DOR: 10, AUC: 0.82). However, only four of the included studies showed downregulated expression of miRNAs. According to previous reports, miRNA may be released by platelet activation during coagulation in serum; therefore, plasma is the preferred sample for studying circulating miRNA markers. Our results are consistent with better diagnostic efficacy in plasma, of course, researchers should pay attention to avoiding hemolysis during the preparation of plasma samples. However, the stages of melanoma may also be a potential source of heterogeneity. The research results of Stark et al. show that the sensitivity of stage IV cohort, can reach 95%, significantly higher than other cohort stage I/II (93%) and stage III (86%) [51]. The stages of melanoma patients included in the study by Armand Labit et al. were IIIc-IVa, [29] the stages of patients included in the study by Li et al. were I-III, [53] and the stages of melanoma patients included in the other studies were I-IV. However, due to the limited number of included studies and the data provided by the original study, subgroup analysis was not able to further explore the sources of heterogeneity, but several studies have shown that miRNA may have better diagnostic efficacy in advanced melanoma, which is a valuable question that was worth we to explore [29]. In addition, according to the results of the QUADAS-2 quality evaluation, the quality of most of the included studies have average quality and all studies are retrospective case control studies. Yao et al., [52]. Mo et al., [50]. Li et al., [31]. Guo et al., [21] and Fogli et al [34] did not mention appropriate selection and exclusion criteria, which may lead to the risk of bias in patient selection, Bai et al., [49] Yao et al., [52] Mo et al., [50] and Li et al [31] have high risk of bias in Index Test may be related to the different implementation process and lack of preset thresholds for testing, so these are potential source of heterogeneity, and more high-quality studies should be included in the future to verify.

According to the subgroup analysis results, the time of collecting patient samples may have an impact on the diagnosis of melanoma, our results are more inclined to collect samples during the diagnosis of melanoma, regardless of surgery and treatment. In view of the complexity of the microRNA regulatory network, the analysis results need to be further tested. The miRNA cluster (Sen: 93%, Spe: 85%, DOR: 73, AUC: 0.95) showed a higher diagnostic efficacy than single miRNA(Sen: 81%, Spe: 78%, DOR: 15, AUC: 0.86). microRNAs can be regulated by multiple genes and target multiple genes. The occurrence and development of melanoma are the result of multi-gene regulation. Therefore, it is imperative for microRNA clusters to become a diagnostic biomarker of melanoma in the future [56]. In the subgroup of miRNA detection methods, only one study used microarrays, while the rest used qRT-PCR, Here, the number of studies needs to be expanded for analysis. Moreover, qRT-PCR is more sensitive than microarrays and is a widely used method for the detection of microRNAs, In addition, it is considered the gold standard technology to verify the results of microarray analysis [57, 58]. Although most previous studies have chosen U6 as the reference gene for normalization, our subgroup analysis showed that other reference genes (Sen: 88%, Spe: 86%, DOR: 44, AUC: 0.93) showed better diagnostic ability than U6(Sen: 87%, Spe: 79%, DOR: 25, AUC: 0.86). Selecting an appropriate reference gene is crucial to the accuracy of qRT-PCR since it is used to normalize the detection results. The most reported endogenous and exogenous genes are hsa-mir-16 (RNU6) and cel-mir-39, respectively. However, the selection of an internal reference gene remains controversial [14]. Moreover, the results of this meta-analysis show that the diagnostic efficacy of plasma(Sen: 0.92, DOR:46, AUC: 0.93) may be higher than that of serum(Sen: 0.81, DOR:19, AUC: 0.88), while the data in whole blood are limited. Interestingly, according our results, there were significant difference in diagnostic ability between the groups with a sample size ≥ 100 and < 100. This may be because a single miRNA may be dysregulated in a various of tumors and not only in patients with melanoma. Additionally, each miRNA may play a different role in tumors, and miRNA clusters can affect tumor initiation and progression via multiple pathways [24].

In addition, in clinical application, the Fagan diagram shows promising results for circulating miRNA as a diagnostic marker for melanoma. We believe that this will help screen potential melanoma patients, improve the diagnostic rate of early melanoma, strive for early risk assessment and intervention measures for atypical mole, multiple moles, and people with family history, continue to expand the sample size and miRNA quantity of research in the future, and optimize the sample collection time and standardized detection process in the overall study, to identify key miRNA molecules for specific diagnosis and prognosis of melanoma.

There are several advantages of this meta-analysis. First, this meta-analysis found circulating miRNAs have high diagnostic value in distinguishing healthy people from patients with malignant melanomas, which provides a new perspective for the development of biomarkers for malignant melanoma diagnosis. Second, this meta-analysis performed a comprehensive miRNA evaluation and conducted subgroup analysis and regression analysis on the influencing factors such as miRNA extraction method, reference, cut-off value, sample sizes, sample type and collection time, to analyze and explore the source of heterogeneity. However, this meta-analysis also has limitations. First, we included a limited number of articles with high overall heterogeneity. Although the analysis showed that studies with a high risk of bias do not overestimate the results, we should still interpret the results with caution. Secondly, the studies we have included are retrospective studies rather than prospective studies, which leads to a higher risk of bias in patient selection and index test domains. Thirdly, due to the limitations of sample sizes and data in the included studies, the diagnostic efficacy of miRNA in the clinical stages and metastasis of melanoma has not been analyzed. In addition, miRNA detection methods should be standardized in future studies. More important is the collection time of blood samples, and our results still need to be further validated. Finally, in order to reduce various biopsychological impacts, prospective sample collection should be widely promoted to obtain more convincing research results.

## Conclusions

In conclusion, existing evidence shows that circulating miRNA has high diagnostic efficacy for melanoma and may be a potential candidate biomarker for the non-invasive diagnosis of melanoma. Additionally, miRNA clusters, collection sample in other time and plasma source samples may have higher diagnostic efficacy. However, our sample size and original literature data were limited and specific subgroup analyses of race, internal reference, stages, and cut-off values, among others, could not be conducted. In the future, high-quality and large-scale research is required to verify our results to find appropriate circulating miRNA diagnostic markers in melanoma.

## Electronic supplementary material

Below is the link to the electronic supplementary material.

Supplementary Material 1

## Data Availability

The datasets used and analyzed during the this study are available from the corresponding author.
